# A Case Report of DVT following the Johnson and Johnson Vaccine against the Novel SARS-CoV-2

**DOI:** 10.1155/2022/1292754

**Published:** 2022-05-17

**Authors:** Ayrton Bangolo, Jeffin Cherian, Mohamed Ahmed, Ali Atoot, Bhavna Gupta, Adam Atoot

**Affiliations:** ^1^Department of Internal Medicine, Hackensack Meridian Health Palisades Medical Center, 7600 River Road, North Bergen, NJ 07047, USA; ^2^Department of Anesthesia, Hackensack University Medical Center, 30 Prospect Avenue, Hackensack, NJ 07601, USA; ^3^Department of Hematology and Oncology, Hackensack Meridian Health Palisades Medical Center, 7600 River Road, North Bergen, NJ 07047, USA

## Abstract

Deep vein thrombosis (DVT) is the formation of a blood clot typically in the deep veins of the extremities. The risk factors for venous thrombosis are primarily related to hypercoagulability, which can be genetic, acquired, or due to immobilization and venous stasis. The Johnson and Johnson (J&J) vaccine developed against the novel SARS-CoV-2 has been linked to more specific cases of thrombosis, associated with low platelet levels similar to that seen in heparin-induced thrombocytopenia. In this case report, we present a female who developed a DVT four days after receiving the J&J vaccine. We propose that the administration of this vaccine may further increase the risk of developing DVTs in patients with previous risk factors, and we hope to use this case to support screening all patients for previous risk factors for thrombosis before administration of the J&J vaccine.

## 1. Introduction

The primary manifestations of venous thrombosis are deep vein thrombosis (DVT) in the extremities and the subsequent embolization to the lungs known as pulmonary embolism (PE), referred to together as a venous thromboembolic disease (VTE). Venous thrombosis occurs due to heritable causes and acquired causes [1]. Of new cases of DVT, up to 30% of patients die within 30 days, owing to the complications associated with pulmonary embolism, and 30% go on to develop recurrent venous thromboembolism within 10 years [1]. The Johnson and Johnson vaccine developed against the novel SARS-CoV-2 has been associated with cavernous venous thrombosis with thrombocytopenia in a few recipients of the vaccine similar in mechanism to heparin-induced thrombocytopenia [2]. Herein, we present a 44-year-old female with previous risk factors for DVT who developed a lower extremity thrombosis four days after receiving the J&J vaccine despite having normal platelet levels. We propose that the administration of this vaccine may further increase the risk of developing DVTs in patients with previous risk factors, and we hope to use this case to support screening all patients prior to the administration of the J&J vaccine.

## 2. Case Presentation

This is a 44-year-old female nonsmoker with a past medical history significant for pulmonary embolism and uterine fibroids on oral contraceptive therapy who presented for evaluation of right lower extremity pain and swelling that started one day before arrival. She denied any recent trauma to the area. The patient reported receiving the Johnson and Johnson vaccine against the novel SARS-CoV-2 five days prior to presentation. One day after receiving the vaccine, the patient reported developing a moderate-grade fever, which was associated with worsening fatigue and myalgias. The patient reported some symptom relief two days after the vaccination. However, on the third day, she began to develop worsening right lower extremity pain which prompted her to seek medical evaluation. Of note, the patient suffered a pulmonary embolism approximately three years ago as a complication of laparoscopic adhesiolysis. No hypercoagulable study was performed at that time, and the patient was started on rivaroxaban for six months. She also reported taking combined oral contraceptive medication for the past two years to control uterine fibroids bleeding.

On physical examination, her vital signs were within normal limits. Erythema was present on the medial aspect of the right thigh and proximal leg as well as diffuse tenderness of the right lower extremity and a positive Homan's sign. On laboratory evaluation, SARS-CoV-2 by polymerase chain reaction was not detected, D-dimer was found to be 1,999 nanograms per milliliter (<500), platelets 299 × 10^*∗*^3 microliters (150–400). A venous Doppler of the right lower extremity revealed acute DVT involving the right femoral vein.

The patient was started on a continuous heparin infusion and underwent a right lower extremity venogram with AngioJet thrombectomy of the right femoral vein. During the venogram, the patient was also found to have significant stenosis of the right external iliac vein and underwent balloon angioplasty. A computed tomography of the abdomen and pelvis without contrast did not reveal any external compression. She reported symptomatic relief shortly after the procedure. The patient was subsequently started on rivaroxaban and the oral contraceptive pills (OCPs) were discontinued. She was discharged with instructions to follow up with hematology as well as gynecology for alternative management of fibroid-associated bleeding.

## 3. Discussion and Conclusion

Although our patient has conventional risk factors for developing a DVT (previous PE and oral contraceptive medication), the timing at which the patient developed the thrombosis raises a few clinical questions: does the vaccine further increase the risk of developing DVT in patients with previous DVTs; Should the vaccine be avoided in patients with other risk factors of DVT? While various societies highlight risk factors leading to the increased risk of developing a DVT, there is an insufficient amount of evidence in the role of the J&J SARS-CoV-2 vaccine itself.

Rare cases of thrombotic events such as cerebral venous sinus thrombosis associated with thrombocytopenia have been reported following vaccination with the J&J vaccine primarily among females <60 years old within the first two weeks of receiving the vaccine. These events were thought to be related to autoantibodies directed against the PF4 platelet antigen, similar to those associated with heparin-induced thrombocytopenia (HIT) [2, 3]. Our patient's right lower extremity thrombosis symptoms occurred 4 days after receiving the vaccine; the patient's HIT testing was not ordered as the platelet level was at baseline and it was not thought that a mechanism similar to HIT was contributing to the patient's present illness. The patient's D-dimer was significantly elevated and a venous Doppler of the right lower extremity confirmed the presence of a DVT.

There are often multiple risk factors of DVT in a given patient at the time of diagnosis [4]. The term unprovoked DVT is used when no environmental provoking events could be identified at the time of DVT. In contrast, the term provoked DVT is used in the setting of an identifiable provoking factor [5]. Risk factors for thrombosis can be seen in up to 80% of cases of DVT and there is often more than one in a given patient [4]. Previous DVT is a major risk factor for recurrent DVT with up to 30% of patients going on to develop at least one more DVT within 10 years [1, 4, 6]. Our patient had a previously provoked DVT three years ago which was secondary to major surgery. Given the patient's previous history of thrombosis and use of OCPs, the administration of this vaccine may have provoked this DVT.

This case report serves to contribute to the growing body of literature regarding the potential adverse effects of the novel J&J vaccine against SARS-CoV-2. By encouraging more clinicians to report similar cases of venous thromboembolic diseases (VTEs), we can help identify a patient population in which this vaccine should be avoided. In addition to current guidelines, we suggest a questionnaire be developed to further screen patients at risk for recurrent DVT.

## Data Availability

All the data generated or analyzed during this study are available from the corresponding author upon request.
